# A Modified Differential Coherent Bit Synchronization Algorithm for BeiDou Weak Signals with Large Frequency Deviation

**DOI:** 10.3390/s17071568

**Published:** 2017-07-04

**Authors:** Zhifeng Han, Jianye Liu, Rongbing Li, Qinghua Zeng, Yi Wang

**Affiliations:** College of Automation Engineering, Nanjing University of Aeronautics and Astronautics, Nanjing 211100, China; ljyac@nuaa.edu.cn (J.L.); lrbing@nuaa.edu.cn (R.L.); zengqh@nuaa.edu.cn (Q.Z.); wangyinuaanrc@163.com (Y.W.)

**Keywords:** bit synchronization, NH code, differential coherent, Beidou System

## Abstract

BeiDou system navigation messages are modulated with a secondary NH (Neumann-Hoffman) code of 1 kbps, where frequent bit transitions limit the coherent integration time to 1 millisecond. Therefore, a bit synchronization algorithm is necessary to obtain bit edges and NH code phases. In order to realize bit synchronization for BeiDou weak signals with large frequency deviation, a bit synchronization algorithm based on differential coherent and maximum likelihood is proposed. Firstly, a differential coherent approach is used to remove the effect of frequency deviation, and the differential delay time is set to be a multiple of bit cycle to remove the influence of NH code. Secondly, the maximum likelihood function detection is used to improve the detection probability of weak signals. Finally, Monte Carlo simulations are conducted to analyze the detection performance of the proposed algorithm compared with a traditional algorithm under the CN0s of 20~40 dB-Hz and different frequency deviations. The results show that the proposed algorithm outperforms the traditional method with a frequency deviation of 50 Hz. This algorithm can remove the effect of BeiDou NH code effectively and weaken the influence of frequency deviation. To confirm the feasibility of the proposed algorithm, real data tests are conducted. The proposed algorithm is suitable for BeiDou weak signal bit synchronization with large frequency deviation.

## 1. Introduction

With the development of Global Navigation Satellite System (GNSS), high-sensitivity receiver design in weak signal environments such as urban canyons, tunnels and dense foliage has become an important issue [1,2,3,4,5,6,7]. In high-sensitivity receivers, the extension of integration time is a typical strategy to enhance the signal-to-noise ratio (SNR) [8]. However, such extension is limited by data bit reversions [9].

The BeiDou Navigation Satellite System is called BeiDou System for short and is scheduled to be completed in 2020. A secondary modulation of the Neumann-Hoffman (NH) code is adopted by the BeiDou System, which plays an important role in spectral separation and narrowband interference protection [10]. However, the periodicity of the transmitted sequence is modified by the NH codes, leading to more polarity changes [11,12]. The data bit rate increases to 1 kbps and the integration time is limited to 1 millisecond.

In the Beidou System, once signal acquisition phase is completed, a bit synchronization phase should be used to detect the position of the data bit edges and NH codes in order to extend the coherent integration time of the tracking loops [13].

The classic algorithm for GPS data bit synchronization is the *histogram method*. It labels the sign changes in consecutive correlation results [14]. If the sign change occurs, the corresponding counter would increase. When the largest counter value exceeds the threshold, the bit boundary is found. Because of its simplicity, the histogram method has been widely used in consumer-level receivers, but it is only adequate for carrier-to-noise ratio (C/N0) above 30 dB-Hz, which is the normal condition for most outdoor GNSS receivers. When the C/N0 is less than 30 dB-Hz, the performance slides fast.

The Viterbi algorithm is also used for bit synchronization [15]. It is adequate for C/N0 even of less than 20 dB-Hz, but its disadvantage is the complicated calculation and increased complexity. For GPS weak signals, the maximum likelihood (ML) bit synchronization method is more popular [16,17]. The ML method calculates the bit energy of all possible navigation bit boundaries. The peak of bit energies corresponds to the bit boundary. This method still has good performance when C/N0 is 20 dB-Hz, but the ML method is sensitive to the frequency deviation. If the frequency deviation is larger than 25 Hz, the ML method is nullified [16,18]. Furthermore, under low C/N0, the frequency deviation is unavoidable and the ML is unreliable.

An efficient differential coherent accumulation algorithm (EDCAA) for weak GPS signal bit synchronization in [19] can eliminate the influence of frequency deviation by adopting the differential coherent value of two consecutive correlation outputs. Authors in [20] proposed a balance differential coherent bit synchronization algorithm for GPS signals, which adopts two complementary differential distances to avoid the unbalanced problem and the accumulation attenuation. Because these two algorithms adopt consecutive correlation outputs to do differential coherent process and the data bit rate of BeiDou is 1 kbps due to NH codes, these algorithms are appropriate for legacy GPS signals but not BeiDou signals.

Because of the NH codes, the data bit rate of BeiDou signals increases to 1 kbps and the integration time is limited to 1 ms. In order to extend the coherent integration time of the tracking loops, a bit synchronization algorithm is necessary to detect the position of the data bit edges and NH codes. In general, for BeiDou weak signals without frequency deviation, the maximum likelihood method can be used for bit synchronization, but considering a frequency deviation larger than 25 Hz, a more appropriate and effective bit synchronization algorithm for BeiDou signals is necessary.

The paper proposes a bit synchronization algorithm for BeiDou weak signals with large frequency deviation. Compared with the previous research in this field, the paper has three novel contributions: (i) the differential coherent method is used to remove the effect of frequency deviation, and the differential delay time is set to be a multiple of bit cycle to remove the influence of NH code; (ii) the maximum likelihood function detection is used to improve the detection probability of weak signals; (iii) Monte Carlo simulations and real data tests are carried out to verify the reliability and performance improvement of the proposed algorithm.

The rest of this paper is organized as follows: Section 2 describes the BeiDou signal model and the conventional bit synchronization method. Section 3 gives detailed descriptions of the proposed algorithm. Section 4 presents the results and analysis of the Monte Carlo simulations. Finally, Section 5 is the conclusion.

## 2. Signal Model and Conventional Algorithm Analysis

### 2.1. Signal Model

**Figure 1:**
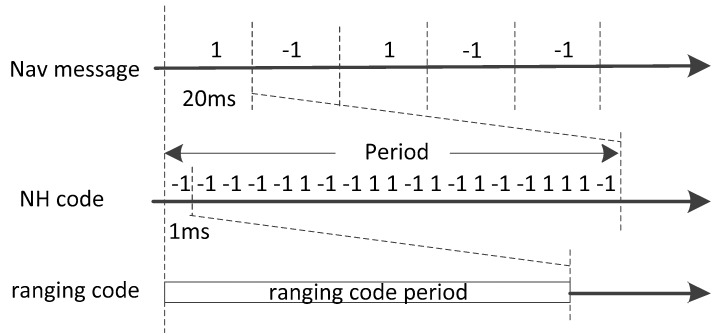
Signal structure of B1I and B2I signal broadcasted by MEO/IGSO and GEO satellites.

Unlike GPS, the space constellation of the BeiDou System consists of Geostationary Earth Orbit (GEO) satellites, Medium Earth Orbit (MEO) satellites and Inclined Geosynchronous Satellite Orbit (IGSO) satellites [21]. The open service signal consists of B1I signal and B2I signal [22]. The signal is composed of the carrier frequency, ranging code and navigation message. The nominal frequency of the B1I signal is 1561.098 MHz, and the nominal frequency of the B2I signal is 1207.140 MHz. The chip rate of the B1I and B2I ranging code is 2.046 Mcps, and the length is 2046 chips. Navigation messages are formatted in D1 and D2 based on their rate and structure. MEO/IGSO and GEO satellites broadcast D1 and D2 navigation messages respectively. For D1 navigation message in format D1 of rate 50 bps, a secondary code of Neumann-Hoffman (NH) code is modulated on ranging code. The period of the NH code is selected to match the duration of a navigation message bit. The bit duration of NH code is the same as one period of the ranging code. The duration of one navigation message bit is 20 milliseconds and the ranging code period is 1 millisecond. Thus the NH code (−1, −1, −1, −1, −1, 1, −1, −1, 1, 1, −1, 1, −1, 1, −1, −1, 1, 1, 1, −1) with length of 20 bits, rate 1 kbps and bit duration of 1 millisecond is adopted. It is modulated on the ranging code synchronously with navigation message bit, as shown in Figure 1. In addition, the rate of D2 navigation message is 500 bps [22].

This paper mainly discusses about the B1I and B2I signal broadcast by MEO/IGSO satellites which is modulated by NH code. The primary symbols and variables used are listed in Table 1.

The signal at the input of a receiver is generally an intermediate frequency (IF) signal, obtained by down-converting, sampling and quantization. The IF signal can be written as follows [22]:

(1) $$s_{IF}(n)\;=AD(nT_{s})H(nT_{s})c(nT_{s}-\tau_{0})\exp[j\cdot 2\pi (f_{IF}+f_{d})nT_{s}+\phi_{0}]$$

where A stands for the carrier amplitude, D and H represent the navigation data and the NH code, and $T_{s}$ is sampling time, c(⋅) represents the ranging code sequence, $\tau_{0}$, $\phi_{0}$, $f_{IF}$ and $f_{d}$ are code propagation delay, initial carrier phase, the carrier IF and Doppler shift.

After signal acquisition, the receiver obtains the approximate incoming frequency ${\hat{f}}_{d,L}$ and ranging code phase ${\hat{\tau }}_{L}$. The locally generated signal sequence is:

(2) $$s_{L}(n)\;=c(nT_{s}-{\hat{\tau }}_{L})\exp[j\cdot 2\pi (f_{IF}+{\hat{f}}_{d,L})nT_{s}]$$

Assuming the coherent integration time is $T_{c}$, the output of the kth coherent integration is [14]:

(3) $$Y(k)=\frac{1}{N_{c}}\sum_{n=kN_{c}}^{(k+1)N_{c}-1}s_{IF}(n)\cdot s_{L}(n)=0.5AD_{k}H_{k}R(\delta \tau )\mathrm{sinc}[\delta f_{d}(k)T_{c}]\exp[j\cdot \phi_{k}+j\pi \delta f_{d}(k)T_{c}]$$

where k denotes the index of the coherent integration interval, $N_{c}=T_{c}/T_{s}$ is the number of samples in the coherent integration time $T_{c}$, $D_{k}$ = ±1 is navigation data, *H_k_* = ±1 is NH code, R(⋅) represents the autocorrelation function of the ranging code, δτ and $\delta f_{d}$ are the errors of code delay estimation and the Doppler shift, $\phi_{k}$ is the initial carrier phase error in the kth coherent integration interval.

### 2.2. Maximum Likelihood Bit Synchronization Method

The maximum likelihood bit synchronization method uses a likelihood function to detect bit boundary locations. The likelihood function used is the sum of the absolute values of cross-correlation function between the prompt correlator output sequence and a window function. In this method, every bit transition can contribute to the detection of the bit boundary, and more bit transitions at the same bit boundary can help to improve the successful synchronization rate (SSR). The ML bit synchronization algorithm is summarized below.

Firstly, there are some parameters that need to be explained. The coherent integration time $T_{c}$ in the tracking stage is 1 ms. The ratio between data bit period and ranging code period is 20, so assume there are 20 possible bit locations. Besides, the length of data sequence to be processed in ML method is N bits. For GPS signals, the 20 ms width window function is defined as:

(4) W(k)=1,  (k=1,2,…20)

For BeiDou signals, the 20 ms width window function is the same with the NH code and is given by:

(5) W(k)=[−1,−1,−1,−1,−1,1,−1,−1,1,1,−1,1,−1,1,−1,−1,1,1,1,−1],  (k=1,2,…20).

The cross-correlation between *Y*(*k*) and *W*(*k*) is given by:

(6) $$C(n,l_{b})=\sum_{k=1}^{20}Y(20n+l_{b}+k)W(k),\;\;\left(n=0,2,\ldots N-1\right),$$

where $l_{b}$ is the initial edge shift of the window function in one bit period. The sum of the absolute values of cross-correlation is:

(7) $$S(l_{b})=\sum_{n=0}^{N-1}\left|C(n,l_{b})\right|$$

Then the ML estimate of the bit boundary is obtained as:

(8) $${\hat{l}}_{b}=\underset{l_{b}\in [1:20]}{\arg\max}S(l_{b})$$

**Figure 2:**
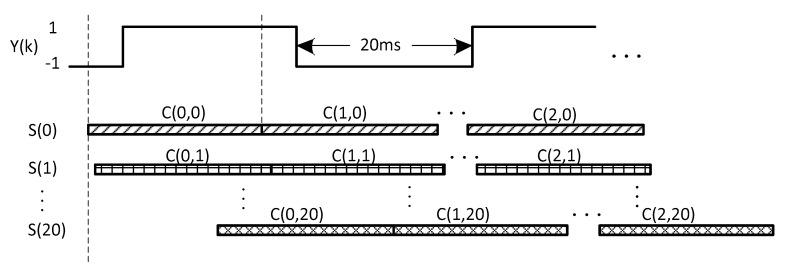
Diagram of the ML bit synchronization method.

Figure 2 is the diagram of the ML bit synchronization method. The maximum sum of the absolute values of cross-correlation corresponding with the real bit boundary can be simply considered as coherent integration of 20 ms and non-coherent integration of N bits (20N ms). Therefore, with longer data sequences or integration times, the performance for weak signals starts to improve.

### 2.3. Influence Factors Analysis

As analyzed above, the maximum sum of the absolute values of cross-correlation can be expressed as:

(9) $$\begin{array}{cc} S({\hat{l}}_{b}) & =\sum_{n=0}^{N-1}\left|C(n,{\hat{l}}_{b})\right|=\sum_{n=0}^{N-1}\left|\sum_{k=1}^{20}Y(20n+{\hat{l}}_{b}+k)\right|\\ & =\sum_{n=0}^{N-1}\left|0.5AD_{k}R(\delta \tau )\mathrm{sinc}[\delta f_{d}(k)T_{coh}]\exp[j\cdot \phi_{k}+j\pi \delta f_{d}(k)T_{coh}]\right| \end{array},$$

where the coherent integration time *T_coh_* is 20 ms.

**Figure 3:**
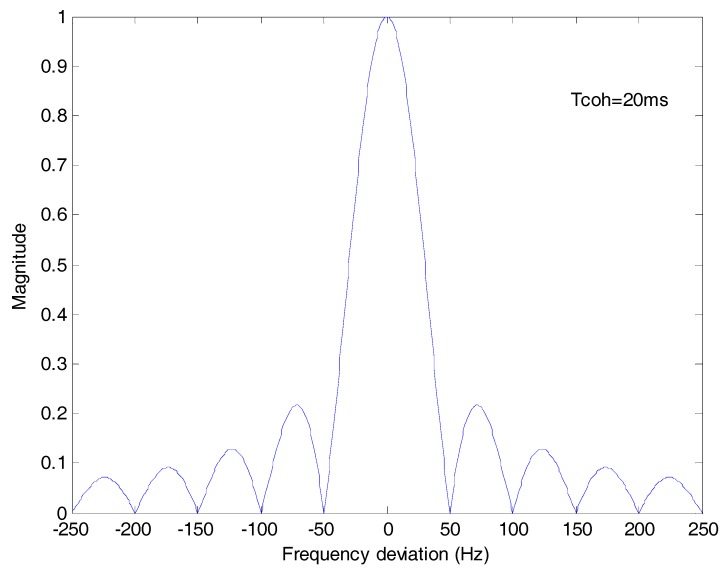
The amplitude attenuation of ML bit synchronization method due to frequency deviation. The coherent integration time *T_coh_* is 20 ms.

In the tracking stage, the code phase offset is small and R(⋅) is close to the peak value, so $\mathrm{sinc}[\delta f_{d}(k)T_{coh}]$ represents the amplitude attenuation of coherent integration due to the frequency deviation. Because the coherent integration time of tracking is 1 ms, the frequency deviation is limited within [−250 Hz, 250 Hz], but the coherent integration time *T_coh_* of the ML method is 20 ms. As shown in Figure 3, the amplitude shall be attenuated to zero with a frequency deviation of an integral multiple of 50 Hz. If the frequency deviation is 25 Hz, the amplitude attenuation 10lg$\mathrm{sinc}[\delta f_{d}(k)T_{coh}]$ shall be −1.9 dB. The results in [16] and [18] show that if the frequency deviation is larger than 25 Hz, the ML method is nullified.

## 3. Proposed Differential Coherent Bit Synchronization Algorithm

### 3.1. Differential Coherent Algorithm

The differential coherent algorithm for bit synchronization was first introduced in [19]. The differential coherent algorithm is implemented on the coherent integration output, by multiplying the present coherent integration result with the delay conjugate of the last one. The mathematical representation of the conventional DC method is:

(10) $$\begin{array}{cc} Q(k) & =Y(k)\cdot Y{\left(k-1\right)}^{*}={\left[0.5AR(\delta \tau )\right]}^{2}\exp(j\cdot \pi \delta f_{d}T_{c})\\ & \times D_{k}D_{k-1}H_{k}H_{k-1}\mathrm{sinc}[\delta f_{d}(k)T_{c}]\mathrm{sinc}[\delta f_{d}(k-1)T_{c}] \end{array}$$

where ( )* denotes the conjugate operation, the coherent integration time $T_{c}$ is 1ms, $\pi \delta f_{d}T_{c}$ is the carrier phase deviation between Y(k) and Y(k−1). The sign of *Q*(*k*) changes due to bit transitions.

Assuming the integration time is 20 ms, the accumulation output of differential coherent values is defined as:

(11) $$\begin{array}{cc} \sum_{k=1}^{u}Q(k) & =\sum_{k=1}^{u}Y(k)\cdot Y{\left(k-1\right)}^{*}={\left[0.5AR(\delta \tau )\right]}^{2}\exp(j\cdot 2\pi \delta f_{d}T_{c})\\ & \times \sum_{k=1}^{u}D_{k}D_{k-1}H_{k}H_{k-1}\mathrm{sinc}[\pi \delta f_{d}(k)T_{c}]\mathrm{sinc}[\pi \delta f_{d}(k-1)T_{c}] \end{array}$$

where *u* is the accumulation time.

For GPS signals, because the sign of product can only change at each bit boundary, there is at most one change in 20 ms and it corresponds with the real bit boundary, but for BeiDou signals, the data bit rate increases to 1 kbps and 10 bit transitions occur in every navigation data bit, so the algorithm for BeiDou signals must be different.

### 3.2. Differential Coherent Bit Synchronization Algorithm

In order to eliminate the influence of frequency deviation, the differential coherent algorithm is combined with the maximum likelihood bit synchronization method. For BeiDou weak signals, bit transitions are more frequent with NH codes. The differential coherent process of two consecutive correlation outputs is not suitable for BeiDou signals.

According to the BeiDou signal structure and NH code modulation, the period of NH code is as long as the duration of a navigation message bit, so $H_{k}=H_{k-20m}$, where m is an integer. In Equation (10), the signs of Q(k) depend on $D_{k}D_{k-1}H_{k}H_{k-1}$. If the delay time of differential coherent is selected to be an integral multiple of 20 ms, $H_{k}H_{k-20m}$ will be equal to 1 and $D_{k}D_{k-20m}H_{k}H_{k-20m}$ will be equal to$D_{k}D_{k-20m}$ which means that the differential coherent result will only depend on transitions of the navigation data and have nothing to do with NH code transitions.

Therefore, in the proposed scheme, the differential coherent delay time is modified to an integral multiple of 20 ms to eliminate the influence of NH code. The concrete steps of the proposed scheme are as follows:

● Step 1. Differential coherent algorithm with long delay time

The delay time of differential coherent is modified to an integral multiple of 20 ms and $H_{k}=H_{k-20m}$. The mathematical representation is:

(12) $$\begin{array}{cc} Z(k) & =Y(k)\cdot Y{\left(k-20m\right)}^{*}={\left[0.5AR(\delta \tau )\right]}^{2}\exp(j\cdot \pi \delta f_{d}mT_{c})\\ & \times D_{k}D_{k-20m}H_{k}H_{k-20m}\mathrm{sinc}[\delta f_{d}(k)T_{c}]\mathrm{sinc}[\delta f_{d}(k-20m)T_{c}]\\ & ={\left[0.5AR(\delta \tau )\right]}^{2}\exp(j\cdot \pi \delta f_{d}mT_{c})\\ & \times D_{k}D_{k-20m}\mathrm{sinc}[\delta f_{d}(k)T_{c}]\mathrm{sinc}[\delta f_{d}(k-20m)T_{c}] \end{array}$$

where *m* is an integral number, $\pi \delta f_{d}mT$ is the carrier phase deviation between Y(k) and Y(k−20m) and it does not change with time.

Because:

(13) $$|\exp(j\cdot \pi \delta f_{d}mT_{c})|=|\cos(\pi \delta f_{d}mT_{c})+j\cdot \sin(\pi \delta f_{d}mT_{c})|=1$$

$\exp(j\cdot \pi \delta f_{d}mT_{c})$ has no effect on the amplitude of Z(k).

$\mathrm{sinc}[\delta f_{d}(k)T_{c}]\mathrm{sinc}[\delta f_{d}(k-20m)T_{c}]$ represents the amplitude attenuation of the differential coherent result due to the frequency deviation. In stable tracking stage, $\delta f_{d}$ changes slightly, so $\delta f_{d}(k)$ can be approximately equal to $\delta f_{d}(k-20m)$ and $\mathrm{sinc}[\delta f_{d}(k)T_{c}]\mathrm{sinc}[\delta f_{d}(k-20m)T_{c}]$ can be approximately equal to $\sin c{\left[\delta f_{d}(k)T_{c}\right]}^{2}$. Here, $T_{c}$ is 1 ms, and the frequency deviation is limited in [−250 Hz, 250 Hz]. Under the worst frequency deviation, the maximum loss 10lg{$\mathrm{sinc}{\left[\delta f_{d}(k)T_{c}\right]}^{2}$} is about −0.9121 dB.

**Figure 4:**
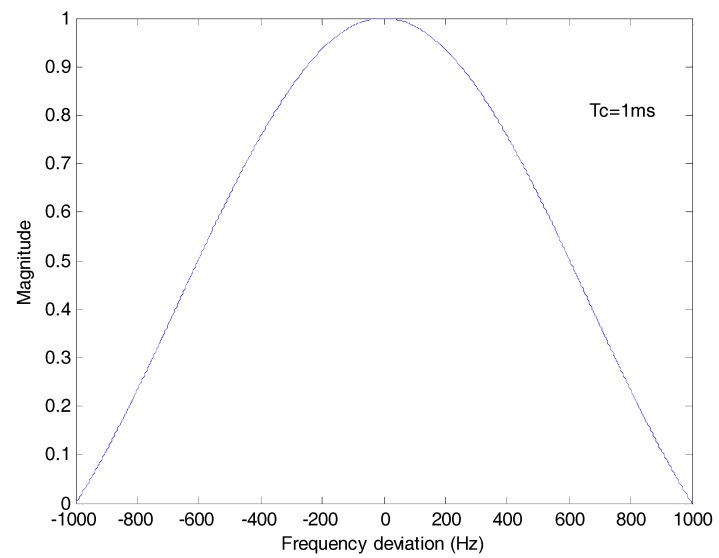
The amplitude attenuation of differential coherent algorithm due to frequency deviation. The integration time *T_c_* is 1 ms.

Figure 4 is the plot of $\mathrm{sinc}{\left[\delta f_{d}(k)T_{c}\right]}^{2}$ which stands for the amplitude attenuation due to the frequency deviation. The frequency deviation effect is greatly eliminated compared with Figure 3.

**Figure 5:**
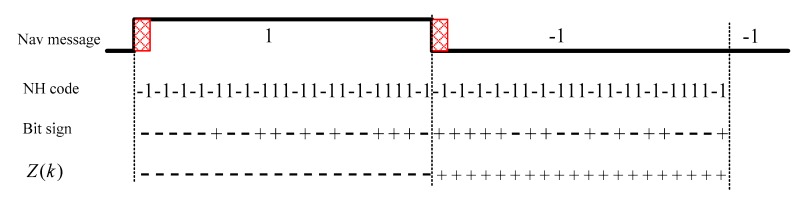
Data signs differential coherent algorithm with long delay time.

Figure 5 shows the signs of data in the differential coherent algorithm with long delay time. It can be seen that after differential coherent process, the signs of *Z*(*k*) only depend on transitions of the navigation data and have nothing to do with NH code.

● Step 2. Maximum likelihood bit synchronization method

Based on differential coherent algorithm with long delay time, the ML bit synchronization method uses the sum of the absolute values of cross-correlation function between *Z*(*k*) and the window function. The 20 ms width window function is:

(14) W(k)=1,  (k=1,2,…20).

The cross-correlation between *Z*(*k*) and *W*(*k*) is given by:

(15) $$C(n,l_{b})=\sum_{k=1}^{20}Z(20n+l_{b}+k)W(k),\;\left(n=0,2,\ldots N-1\right).$$

The sum of the absolute values of cross-correlation is:

(16) $$S(l_{b})=\sum_{n=0}^{N-1}\left|C(n,l_{b})\right|.$$

Then the ML estimate of bit boundaries is obtained as:

(17) $${\hat{l}}_{b}=\underset{l_{b}\in [1:20]}{\arg\max}S(l_{b}).$$

The maximum sum of the absolute values of cross-correlation can be expressed as:

(18) $$\begin{array}{cc} S({\hat{l}}_{b}) & =\sum_{n=0}^{N-1}\left|C(n,{\hat{l}}_{b})\right|=\sum_{n=0}^{N-1}\left|\sum_{k=1}^{20}Z(20n+{\hat{l}}_{b}+k)\right|\\ & =\sum_{n=0}^{N-1}\left|{\left[0.5AR(\delta \tau )\right]}^{2}\exp(j\cdot \pi \delta f_{d}mT_{c})\sum_{k=1}^{20}D_{k}D_{k-20m}\mathrm{sinc}[\delta f_{d}(k)T_{c}]\mathrm{sinc}[\delta f_{d}(k-20m)T_{c}]\right| \end{array},$$

here, $\sum_{k=1}^{20}\sin c[\delta f_{d}(k)T_{c}]\sin c[\delta f_{d}(k-20m)T_{c}]$ represents the frequency deviation effect of the proposed algorithm and $\sin c[\delta f_{d}(k)T_{coh}]$ in Equation (9) represents the frequency deviation effect of the conventional ML algorithm. In order to compare the frequency deviation effects of the two algorithms, we should make approximations to $\sum_{k=1}^{20}\sin c[\delta f_{d}(k)T_{c}]\sin c[\delta f_{d}(k-20m)T_{c}]$. Because $\delta f_{d}$ changes slightly in stable tracking stage, $\delta f_{d}(k)$ can be approximately equal to $\delta f_{d}(k-20m)$ and $\sin c[\delta f_{d}(k)T_{c}]\sin c[\delta f_{d}(k-20m)T_{c}]$ can be approximately equal to $\sin c{\left[\delta f_{d}(k)T_{c}\right]}^{2}$ as:

(19) $$\sum_{k=1}^{20}\mathrm{sinc}[\delta f_{d}(k)T_{c}]\mathrm{sinc}[\delta f_{d}(k-20m)T_{c}]\approx \sum_{k=1}^{20}\left\{\mathrm{sinc}[\delta f_{d}(k)T_{c}\right]{\}}^{2}\approx 20{\left\{\mathrm{sinc}[\delta f_{d}(k)T_{c}]\right\}}^{2}.$$

**Figure 6:**
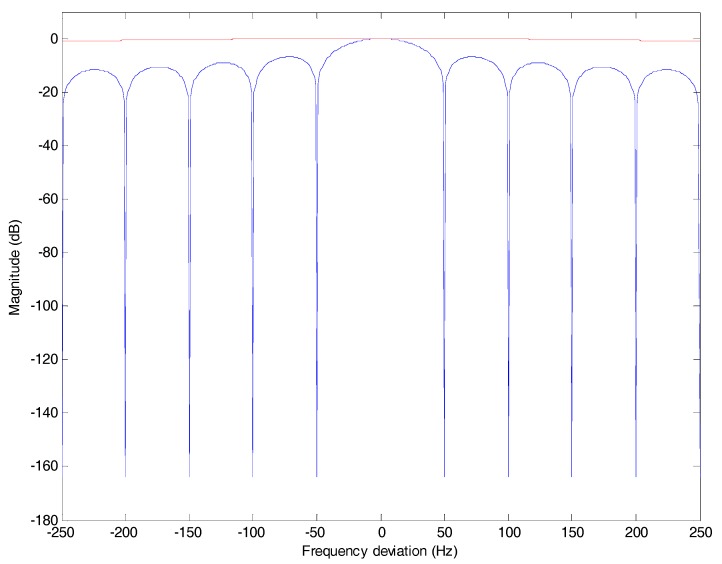
The amplitude attenuations of ML and DCML bit synchronization algorithms due to frequency deviation. The blue line is for ML and the red line is for DCML.

In Figure 6, the red line stands for 10 × lg($20{\left\{\mathrm{sinc}[\delta f_{d}(k)T_{c}]\right\}}^{2}$) and the blue line stands for 10 × lg($\mathrm{sinc}[\delta f_{d}(k)T_{coh}]$). It can be seen that the absolute value of the former is much smaller than the latter especially with a frequency deviation of an integral multiple of 50 Hz, because *T_c_* is 1 ms and *T_coh_* is 20 ms. 

Therefore, $\sum_{k=1}^{20}\mathrm{sinc}[\delta f_{d}(k)T_{c}]\mathrm{sinc}[\delta f_{d}(k-20m)T_{c}]$ in the Equation (19) is much smaller than $\mathrm{sinc}[\delta f_{d}(k)T_{coh}]$ in the Equation (9). Therefore, the proposed bit synchronization algorithm should outperform the conventional ML algorithm with a frequency deviation of an integral multiple of 50 Hz. As above, the proposed bit synchronization algorithm eliminates the influence of frequency deviation by adopting differential coherent algorithm and eliminates the influence of NH code by modifying the differential delay time to an integral multiple of 20 ms.

## 4. Simulation Results and Analysis

### 4.1. Monte Carlo Simulations

**Figure 7:**
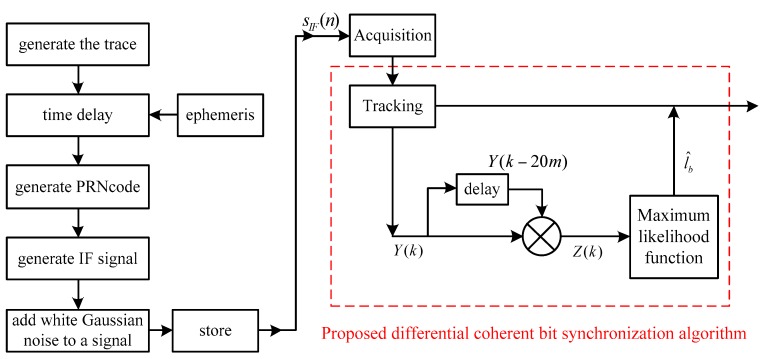
The diagram of the platform for Monte Carlo simulations.

To achieve a comprehensive assessment of the proposed bit synchronization scheme, Monte Carlo simulations are carried out. The simulation platform is shown in Figure 7, which consists of BeiDou B1I IF signal generation and software receiver baseband process. The parameters used in Monte Carlo simulations are provided in Table 2.

The concrete steps of the BeiDou B1I IF signal generation simulation are as follows.

(1) Position and velocity of satellites are resolved based on stored ephemeris parameters.
(2) The time delay variable is calculated according to the position and velocity of satellites and the trace.
(3) On the basis of BeiDou signal structure, carrier, ranging code and NH code are generated.
(4) The BeiDou IF signal is generated according to the time delay variable and signal modulation.
(5) Additional white Gaussian noise is generated according to the CN0 settings.
(6) Every sampled point of the signal is quantized and stored in two bits.

**Figure 8:**
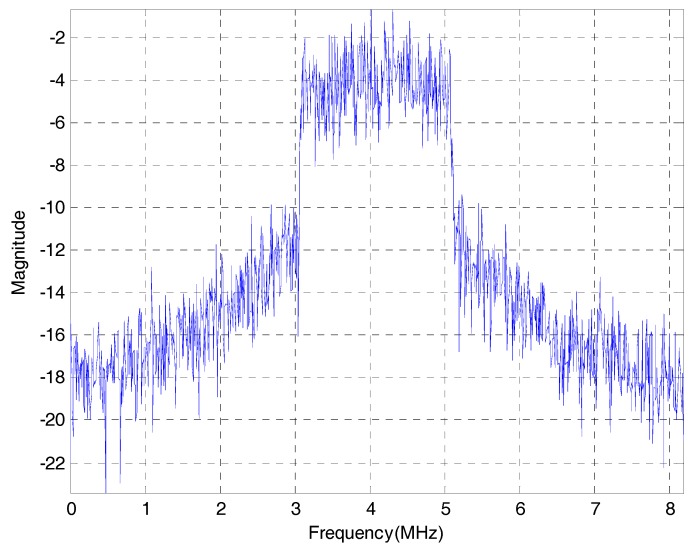
Signal spectrum distribution of the BeiDou B1I IF signals.

The signal spectrum distribution of the IF samples is shown in Figure 8.

In the software receiver section, the proposed differential coherent bit synchronization algorithm is after conventional signal acquisition and tracking. Repeated acquisition based on 1~2 ms of coherent integration is used for signal acquisition, and DLL and PLL are used for signal tracking.

To prove the reliability of the proposed algorithm, tests are done to evaluate the sensitivity of different CN0s. Using Monte Carlo simulations, additional white Gaussian noise is generated for each trial, and 1000 trials are used for each probability. Monte Carlo simulations of different algorithms are carried out for comprehensive evaluation.

**Figure 9:**
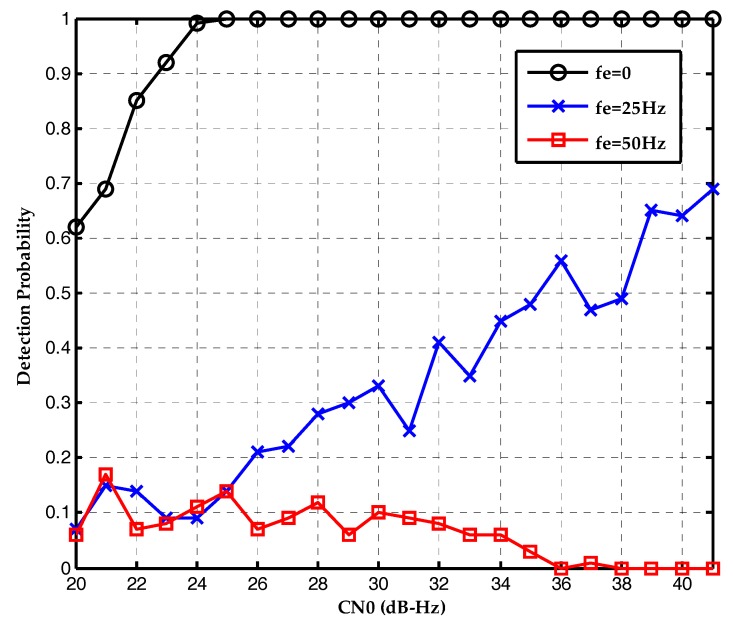
Detection probabilities of the ML bit synchronization algorithm under the CN0s of 20~40 dB-Hz and different frequency deviations.

The first simulation tests the effect of frequency deviation on the conventional maximum likelihood bit synchronization algorithm. The detection probability is plotted as a function of the CN0s in Figure 9. Without frequency deviation, the conventional ML bit synchronization algorithm has good performance in weak signal environment. The detection probability is 0.92 at CN0 of 23 dB-Hz. However, with the increase of frequency deviation, the detection probability attenuates seriously. When the frequency deviation is 50 Hz, the ML method is nullified.

**Figure 10:**
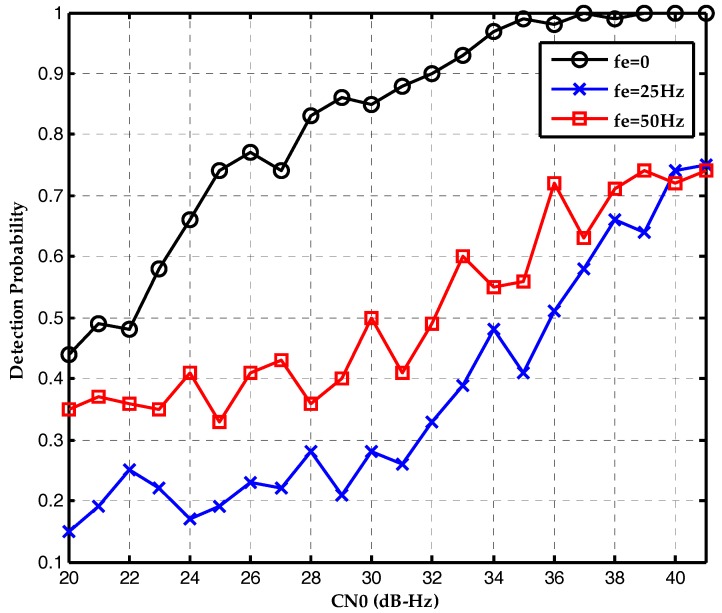
Detection probabilities of the DCML bit synchronization algorithm under the CN0s of 20~40 dB-Hz and different frequency deviations.

Therefore, $\sum_{k=1}^{20}\mathrm{sinc}[\delta f_{d}(k)T_{c}]\mathrm{sinc}[\delta f_{d}(k-20m)T_{c}]$ in the Equation (19) is much smaller than $\mathrm{sinc}[\delta f_{d}(k)T_{coh}]$ in the Equation (9). Therefore, the proposed bit synchronization algorithm should outperform the conventional ML algorithm with a frequency deviation of an integral multiple of 50 Hz. The second simulation evaluates the effect of frequency deviation on the proposed bit synchronization algorithm. The detection probability is plotted as a function of the CN0s in Figure 10. With the increase of frequency deviation, the detection probability attenuates too, but not seriously. When the frequency deviation is 50 Hz, the detection probability is 0.72 at CN0 of 36 dB-Hz.

**Figure 11:**
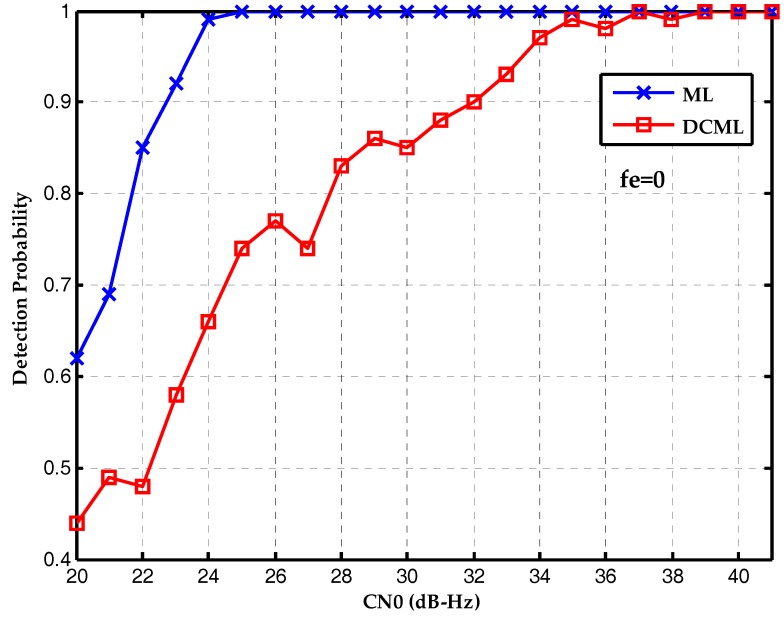
Detection probabilities of ML and DCML bit synchronization algorithms under the CN0s of 20~40 dB-Hz with a frequency deviation of 0 Hz.

**Figure 12:**
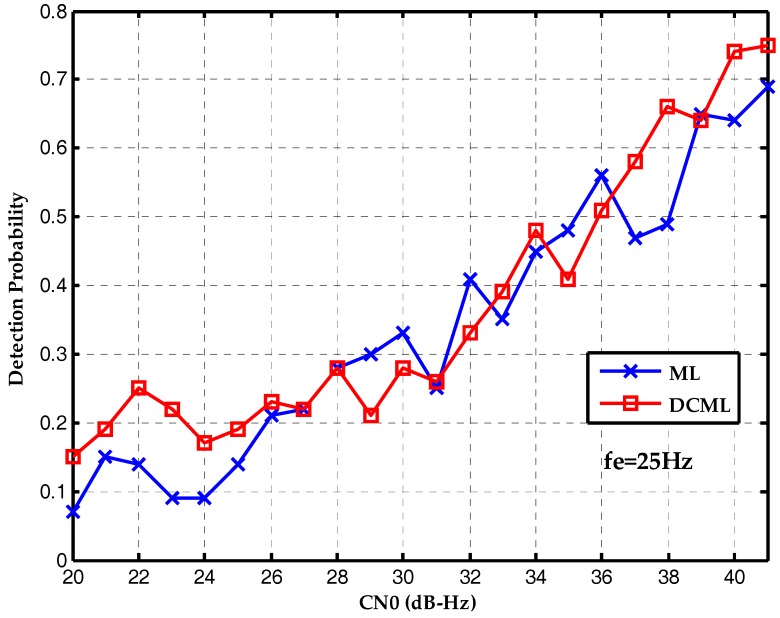
Detection probabilities of ML and DCML bit synchronization algorithms under the CN0s of 20~40 dB-Hz with a frequency deviation of 25 Hz.

**Figure 13:**
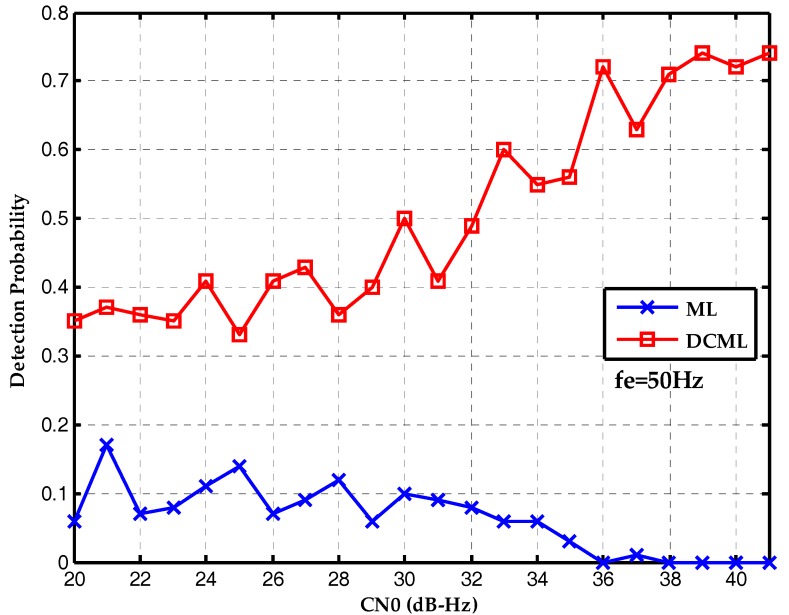
Detection probabilities of ML and DCML bit synchronization algorithms under the CN0s of 20~40 dB-Hz with a frequency deviation of 50 Hz.

Finally, the performances of two algorithms are compared with different frequency deviations. Figure 11 is the detection probability without frequency deviation. The conventional maximum likelihood method outperforms the proposed bit synchronization algorithm which has gain attenuation. Figure 12 is the detection probability with a frequency deviation of 25 Hz and the performances of two algorithms are about the same. Then, Figure 13 is the detection probability with a frequency deviation of 50 Hz. It is clear that the proposed bit synchronization algorithm outperforms the conventional maximum likelihood method and the detection probability of the proposed bit synchronization algorithm is always higher than the conventional maximum likelihood method.

The results above show that the detection probability of the traditional maximum likelihood method attenuates seriously with the increase of frequency deviation and the ML method is even nullified when the frequency deviation is 50 Hz. By contrast, the detection probability of the proposed bit synchronization algorithm does not attenuate very seriously with the increase of frequency deviation. The detection probability of the proposed bit synchronization algorithm is always higher than the conventional maximum likelihood method when the frequency deviation is 50 Hz. Therefore, the proposed bit synchronization algorithm outperforms the conventional maximum likelihood method with large frequency deviation which is consistent with the theory analysis in Section 3.2.

### 4.2. Real Data Tests

**Figure 14:**
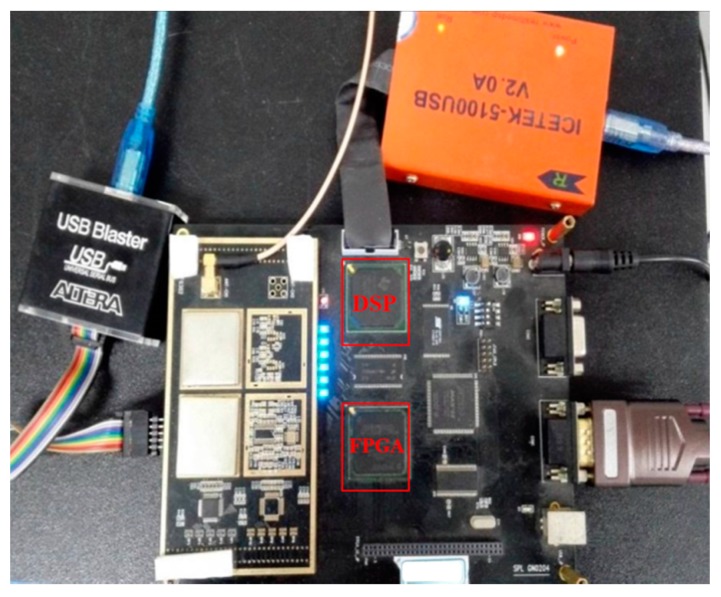
The BeiDou B1I receiver test platform.

To confirm the feasibility of the proposed algorithm, real data tests are conducted. The BeiDou B1I receiver used was developed by the Navigation Research Center, Nanjing University of Aeronautics and Astronautics (NRC, NUAA) and the hardware structure was developed by Shanghai Yuzhi. As shown in Figure 14, the receiver test platform consists of a DSP-TMS320C6713B and an FPGA-EP4CE115F23, and an AD8347 is used as the quadrature down-conversion mixer. The parameters used are provided in Table 3. The antenna is placed on the roof of College of Automation Engineering building of NUAA and the signal is processed in real time.

In the BeiDou B1I receiver, repeated acquisition based on 1~2 ms of coherent integration is used for signal acquisition. DLL and PLL assisted by FLL are used for signal tracking. The proposed differential coherent bit synchronization algorithm is processed after signal acquisition and tracking.

**Figure 15:**
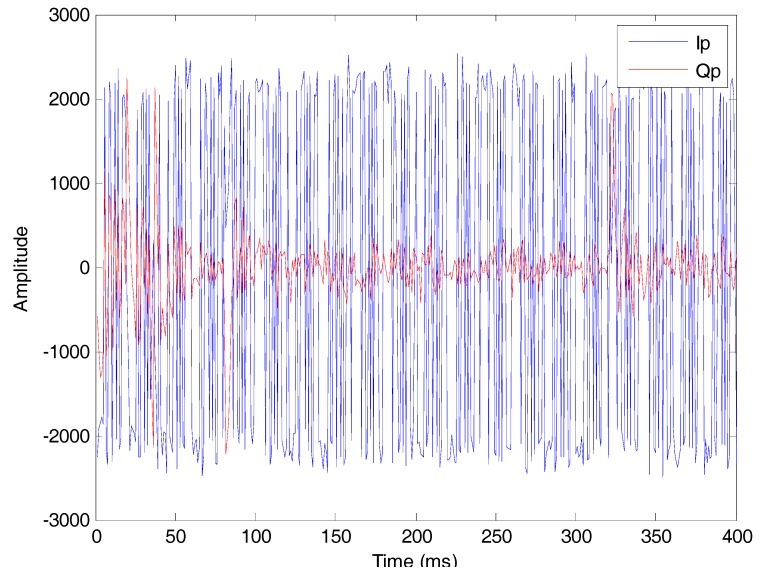
The tracking result of satellite #9.

The number of visible satellites is ten, including five GEO, three IGSO and two MEO. Take satellite #9 as an example and the result of tracking loop is shown in Figure 15.

**Figure 16:**
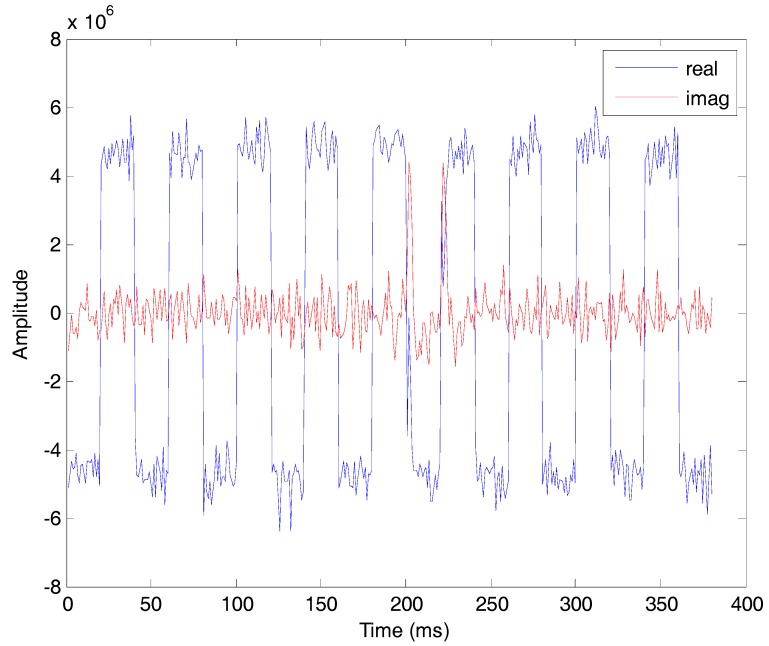
The differential coherent result of satellite #9.

**Figure 17:**
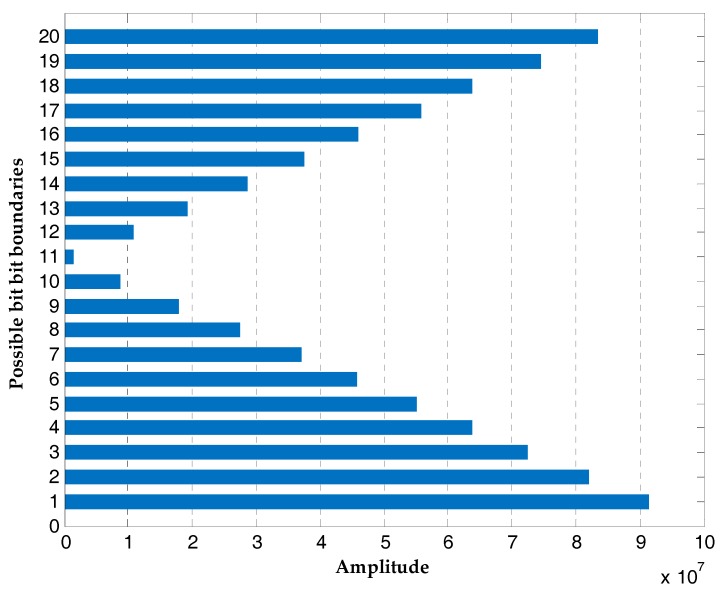
The amplitudes of DCML algorithm at all possible bit boundaries.

The proposed bit synchronization algorithm is processed based on the coherent integration results of tracking loops. Figure 16 is the result of differential coherent algorithm with a delay time of 20 ms and the signs are consistent with transitions of the navigation data. The amplitudes of DCML algorithm at all possible bit boundaries are shown in Figure 17 and the bit boundary estimation is one. After bit synchronization, the bits are decoded and position calculation is realized.

## 5. Conclusions

In order to realize bit synchronization for BeiDou weak signals with large frequency deviation, a bit synchronization algorithm based on differential correlation and maximum likelihood is proposed. Firstly, the differential correlation is used to remove the effect of frequency deviation, and the differential delay time is set to be a multiple of bit cycle to remove the influence of NH code. Secondly, the maximum likelihood function detection is used to improve the detection probability of weak signals.

Monte Carlo simulations are carried out to analyze the detection performance of the proposed algorithm compared with a traditional algorithm under the CN0s of 20~40 dB-Hz and different frequency deviations. The results show that the proposed algorithm outperforms the traditional method when the frequency deviation is 50 Hz. This algorithm can remove the effect of BeiDou NH code effectively and weaken the influence of frequency deviation. This algorithm is suitable for BeiDou weak signal bit synchronization with large frequency deviation. In addition, this method is applicable to BeiDou signals and it is also applicable to GPS, Galileo and other satellites navigation systems.

## Figures and Tables

**Table 1:** Primary symbols and variables.

Variable	Meaning
$s_{IF}(n)\;$	received IF signal
A	carrier amplitude
D(n)	navigation data
H(n)	NH code
c(⋅)	ranging code
$T_{s}$	sampling time
$f_{IF}$	carrier IF frequency
$f_{d}$	Doppler frequency shift
$\tau_{0}$	code propagation delay
$\phi_{0}$	initial carrier phase
$s_{L}(n)\;$	locally generated signal
${\hat{\tau }}_{L}$	ranging code phase of local signal
${\hat{f}}_{d,L}$	carrier frequency shift of local signal
*Y*(*k*)	output of the kth coherent integration
$T_{c}$	integration time
$N_{c}$	the number of samples in the integration time
R(⋅)	autocorrelation function of the ranging code
δτ	code phase estimation error
$\delta f_{d}$	Doppler frequency estimation error
$\phi_{k}$	carrier phase estimation error
W(k)	window function
$C(n,l_{b})$	cross-correlation between *Y*(*k*) and *W*(*k*)
$S({\hat{l}}_{b})$	sum of the absolute values of cross-correlation
$l_{b}$	estimate of the bit boundary
$T_{coh}$	coherent integration time
*Q*(*k*)	differential coherent values
*Z*(*k*)	Differential coherent values with long delay time

**Table 2:** Parameters used in the Monte Carlo simulations.

Sections	Parameters	Values
Signal Generation	IF frequency	4.1304 MHz
	sampling frequency	16.3676 MHz
	visible satellites	6, 7, 11 and 12
	CN0	20~40 dB-Hz
Software Receiver	DLL bandwidth	2 Hz
	PLL bandwidth	40 Hz
	frequency deviation	0, 25, 50 Hz
	differential coherent delay time	20 ms
	differential coherent integration time	40 ms

**Table 3:** Parameters used in real data tests.

Parameters	Values
IF frequency	4.098 MHz
Sampling frequency	62 MHz
DLL bandwidth	5 Hz
PLL bandwidth	20 Hz
FLL bandwith	40 Hz
Differential coherent delay time	20 ms
Differential coherent integration time	40 ms
